# Health System- and Payer-Level Decision-Making Processes Influencing the Adoption and Sustainability of Patient-Facing Digital Health Tools: Qualitative Study

**DOI:** 10.2196/97536

**Published:** 2026-08-14

**Authors:** Mallory Herzog, Linda S Park, Andrew Quanbeck

**Affiliations:** 1 Department of Family Medicine and Community Health, School of Medicine and Public Health, University of Wisconsin–Madison, 610 N Whitney Way, STE 200, Madison, WI, 53705, United States

**Keywords:** patient-facing digital health, implementation science, learning health systems, organizational decision-making, qualitative research

## Abstract

**Background:**

Patient-facing digital health tools such as mobile health apps, wearables, and digital therapeutics have expanded rapidly and show promise for improving chronic disease management. Despite increasing evidence of effectiveness, health systems and payers continue to face challenges integrating these tools into routine care.

**Objective:**

This study examined the decision-making processes of health system and payer leaders regarding the adoption and sustainability of patient-facing digital health tools within their organizations.

**Methods:**

We conducted semistructured interviews between August 2025 and December 2025 with nine senior leaders from a large Midwestern academic health system and affiliated payer organizations, including a provider-owned health plan and a state Medicaid program. Interviews explored digital health adoption decisions, perceived value and fit, barriers, and sustainability considerations, focusing on the adoption of an evidence-based mobile health intervention for alcohol use disorder as a use case. Transcripts were analyzed using thematic analysis with inductive and deductive coding.

**Results:**

Four decision-making mechanisms shaped adoption and sustainability decisions: prioritization under organizational constraint, risk mitigation, operational fit, and value determination. These mechanisms describe how leaders navigate limited organizational capacity, reduce uncertainty, and protect against clinical, financial, and operational risks, assess whether tools can integrate within existing clinical and technical systems, and determine whether anticipated and measurable benefits justify adoption and continued organizational support.

**Conclusions:**

Adoption and sustainability of patient-facing digital health tools are shaped by dynamic organizational decision-making processes that often remain invisible to patients, clinicians, researchers, and developers. Making these processes visible may help better align digital health tools with the realities of the health care system to support implementation.

## Introduction

Digital health tools have emerged as potentially scalable solutions to expand access to chronic disease self-management by extending support beyond traditional clinical settings. In this study, patient-facing digital health tools may include mobile health (mHealth) apps, web-based platforms, patient portals, or digital therapeutics that support self-management of health, access to health information, or engagement with care [1-3]. Recently, the mHealth market has expanded to more than 300,000 apps spanning general wellness to disease-specific management, alongside increasing approvals for prescription digital therapeutics and wearables [3]. In parallel, a growing body of evidence supports the effectiveness of patient-facing digital health tools, with their value increasingly recognized by health systems and payers [4-6].

Coinciding with the expansion of patient-facing digital health tools, clinicians and patients report feeling inundated by a saturated third-party marketplace in which individuals can access tools through app stores, a phenomenon commonly known as “app fatigue” [7]. Physicians have also noted a barrier to offering digital health tools in care due to the absence of institutional technical validation and support, as these direct-to-consumer tools operate outside of the health system infrastructure and raise concerns about privacy, security, efficacy, safety, and quality [8-10].

These concerns may be addressed by institutional adoption and endorsement of digital tools, defined in this study as the organizational decision to support or integrate a tool into care delivery. Adoption can signal quality, align tools with workflows, and establish responsibility for oversight [9]. Institutional adoption of a digital tool may also increase patient uptake and trust in the tool as they are often perceived as more credible than those used independently [11]. Concurrently, institutional adoption may introduce governance and liability considerations for the adopting organization [9]. In response to these trade-offs, recent reports indicate that health systems and payers are moving toward more selective adoption of digital health technologies, emphasizing robust clinical evidence, integration into clinical workflows and data systems, and clearer reimbursement pathways [6]. Still, there remains a struggle to sustain patient-facing digital health tools within health care systems and payer organizations, meaning the maintenance of organizational support and use of these tools after initial adoption, which reflects persistent challenges in implementation [12,13].

To support adoption decisions, numerous evaluation frameworks have been developed to assess digital health tools across domains such as clinical effectiveness, data privacy, usability, and value [12,14-17]. In parallel, implementation science frameworks such as the Exploration, Preparation, Implementation, Sustainment model conceptualize how evidence-based interventions are introduced and maintained within health care systems [18]. Together, these frameworks provide a useful structure for evaluating digital health tools and understanding implementation processes that are common across many new health interventions. However, patient-facing digital health tools also introduce distinct implementation challenges because they function not only as clinical interventions but also as technology products. This, therefore, creates challenges related to data governance, privacy and security, interoperability, software maintenance, and how to deal with patient-generated data [19].

Digital health evaluation frameworks also face several challenges in practice. These include balancing objective and subjective criteria, engaging end users, remaining current alongside rapid technological evolution, incorporating real-world usage and effectiveness measurements, and accounting for adoption as an ongoing process [20,21]. Additionally, adoption decisions within large, complex health systems and payer organizations require multistakeholder input and face dynamic financial, operational, and technical constraints that may be difficult to capture within a standardized framework [12].

Consistent with these challenges, recent studies have examined cross-stakeholder perspectives on digital health tool adoption, illustrating misalignment across digital health stakeholder groups and identifying opportunities for improved communication and coordination between groups to support adoption [2,22]. Within health systems specifically, existing literature has largely provided clinician or implementer experiences as a descriptive understanding of digital tool implementation [12,23,24]. Comparatively, studies focused on payer digital health decision-making are sparse and have predominantly addressed reimbursement considerations [25,26]. Together, prior studies have provided valuable insight into stakeholder perspectives and implementation contexts but have insufficiently captured how digital health tool decisions are navigated within and across organizations.

To examine this gap, we used the Tula (Sanskrit for “balance”) app, a patient-facing mHealth app for individuals with mild-to-moderate alcohol use disorder (AUD), as an illustrative use case to explore how a specific digital tool might be considered for implementation and to examine broader organizational decision-making around digital health [27,28]. A recent hybrid effectiveness-implementation trial demonstrated that Tula reduced heavy drinking days across self-guided, peer-supported, and health coach–supported models, highlighting its potential to be offered as an effective and scalable intervention for AUD [28]. Building on this work, a qualitative study examined perceptions among patient participants and implementers of Tula, identifying areas of fit and misfit of the app [29]. While this prior qualitative study provided insight into end-user and implementer perspectives, a gap remained in the broader organizational-level perspectives of health system leaders and payers.

Systematically examining organizational decision-making aligns with the multilayer framework of the health care system provided by the National Academy of Engineering that places patient experiences nested within care teams, an organizational structure, and a regulatory and policy environment [30]. Decisions made at the system level remain cloudy to patients, clinicians, researchers, and digital tool developers, yet they are a critical determining factor in which tools become integrated into practice. In this study, we use decision-making processes to refer to the criteria, trade-offs, and organizational considerations leaders use when evaluating patient-facing digital health tools. This qualitative study addresses this gap by making visible how senior leaders within a health system and its associated public and private payer organizations actually navigate adoption and sustainability decisions for patient-facing digital health tools, using Tula as an example use case to ground these processes.

## Methods

### Study Design

**Figure 1.**
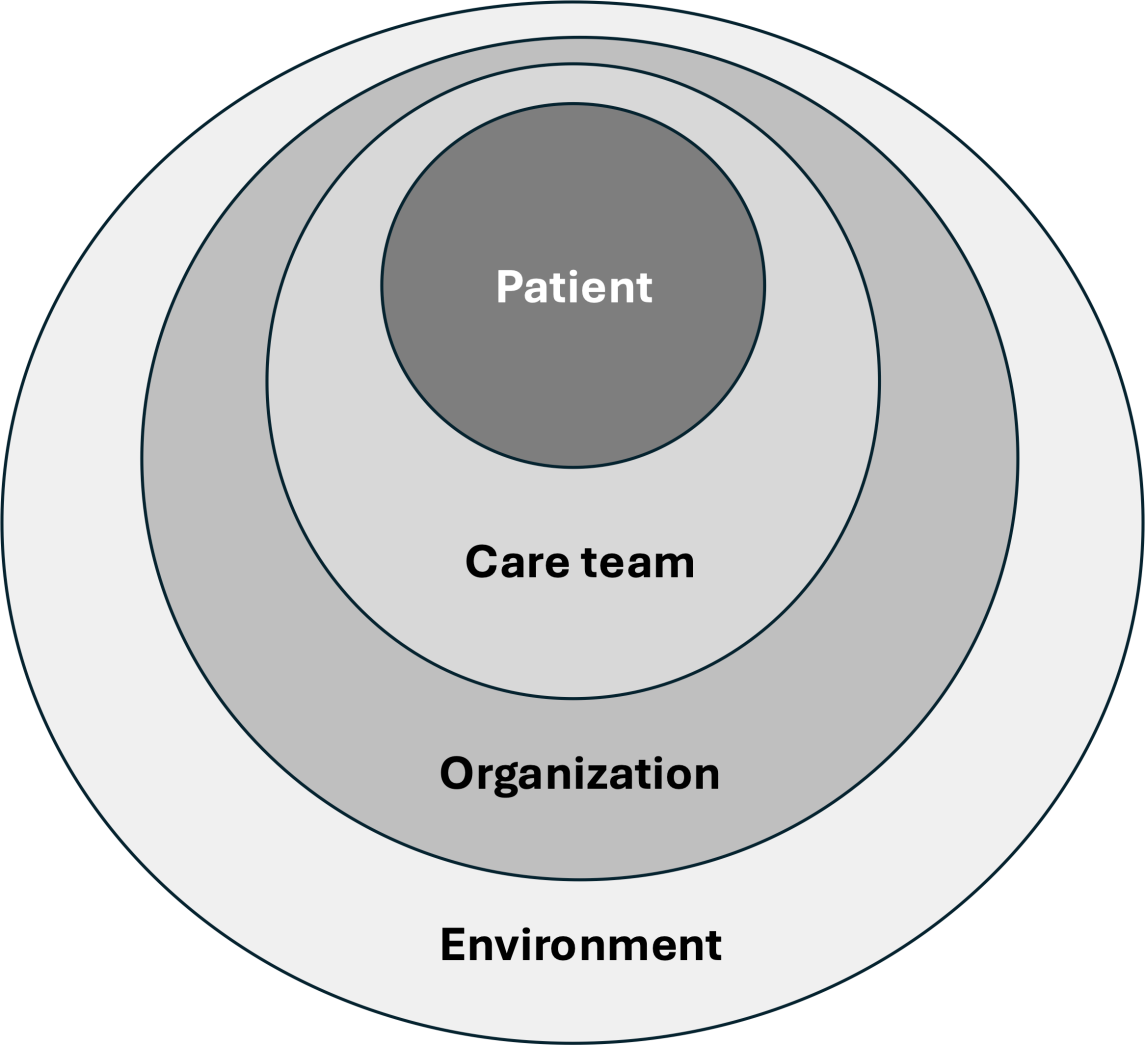
Conceptualization of health system informing study design. Adapted from the National Academy of Medicine and the Institute of Medicine model of health care delivery.[30].

This qualitative study included a series of 60-minute semistructured interviews with senior leaders from a large academic health system and its associated private and public payers to evaluate how digital tool adoption and sustainability decisions are made. Guided by the multilayer framework from the National Academy of Engineering described in the *Introduction*, this study focused on decision-making in the health system at the organizational level and among payers within the broader environmental layer (Figure 1).

Interviews were guided by a standardized interview template that included the following domains: role and organizational context; current AUD management; digital tool adoption decisions; perceived value and fit; barriers to adoption; and sustainability and long-term fit. These domains provided consistency across interviews, while some questions were tailored to fit the participants’ roles and organizational contexts. The Tula app was used as a grounding example during interviews, but participants were also asked to reflect on broader experiences with patient-facing digital health tools. Additionally, to mitigate potential bias and enhance trustworthiness, MH avoided leading participants toward predetermined conclusions and adjusted follow-up questions during interviews to allow participants to describe decision-making processes in their own terms. The full interview guide is provided in Multimedia Appendix 1.

### Recruitment

This study was conducted within a large, Midwestern integrated academic health system associated with a public research university that uses a value-based care model situated in the context of a learning health system and accountable care organization. Nine interview participants working within this health system and its affiliated payer organizations were identified through purposive and snowball sampling [31]. Initial participants were identified through professional and department networks. Following interviews, participants were asked to recommend additional individuals who met the eligibility criteria of holding a health system or payer organizational role with decision-making responsibilities surrounding digital health tools. Efforts were made to include a diversity of decision-making roles across the health system and its associated payers, and sampling continued until representation across decision roles was achieved (Table 1).

**Table 1.** Study participants by organization type and decision-making role.

Organization type	Participants, n	Participant roles
Health system	7	Clinical leader (n=4); digital leader (n=2); informatics leader (n=1)
Payer	2	Provider-owned health plan leader (n=1); state Medicaid leader (n=1)

Participant roles were categorized based on role responsibilities described during interviews and used primarily as analytic descriptors. Clinical roles reflected responsibility for care delivery and clinical operations. Digital roles reflected responsibility for digital health strategy, evaluation, and implementation. Informatics roles reflected responsibility for clinical information systems and data governance. Payers were categorized separately by payer type (provider-owned health plan and Medicaid). To preserve confidentiality, specific role types were not reported, and participants were assigned anonymized identifiers based on organization type: health system leaders (HSL1-HSL7) and payer leaders (PL1-PL2), which are used when presenting quotes.

### Data Analysis

All interviews were audio recorded via Zoom (version 5.11.3; Zoom Video Communications) and transcribed using the platform’s automated transcription feature. Recordings were subsequently reviewed alongside transcripts to ensure accuracy and make corrections as needed.

Interview transcript data were analyzed with a thematic analysis approach using both inductive and deductive coding elements [32]. Transcripts were coded using Dedoose (version 10.0.25; SocioCultural Research Consultants), a cross-platform application for qualitative and mixed methods research.

Initial deductive domains were informed by the interview template used to guide each interview, creating a preliminary codebook. Primary coding was conducted by the lead researcher (MH). A second qualitative researcher (LSP) reviewed and independently coded 2 transcripts (1 from a health system role and 1 from a payer role) to support intercoder reliability. LSP and MH met to compare code definitions and discuss interpretive discrepancies. After discrepancies were resolved through group consensus, the preliminary codebook was iteratively refined to create a codebook that was applied to all transcripts. Within and across these domains, inductive codes were developed that captured emerging concepts and patterns in the data. The codebook then continued to be iteratively refined throughout analysis using comparison across transcripts until we were able to establish clear definitions for each code.

Following coding, related codes were grouped into higher-level categories and compared across transcripts to identify broader patterns across the data. These groupings then informed the development of themes, which were iteratively refined through team discussion, comparison across participant roles, and review of analytic memos that documented coding decisions, emerging interpretations, and reflexive notes.

During analysis, AUD-specific considerations, such as outcome measurement using the Alcohol Use Disorders Identification Test-Consumption (AUDIT-C) [33], treatment access gaps, or clinical limitations in AUD management, were examined in relation to the broader decision-making processes described by participants. These considerations informed parts of the analysis but were reflected within the broader mechanisms rather than forming separate themes.

Sampling and analysis continued until the team determined that the interviews had reached sufficient analytic depth for the study aims (saturation). Among health system participants, code stability was reached relatively early, with later interviews reinforcing existing concepts while adding role-specific examples. Across payer interviews, overlap with the health system interviews in the overarching decision-making mechanisms provided additional support for the analytic depth of the findings. However, because the 2 payer participants represented different payer contexts, including a state Medicaid program and a provider-owned health plan, payer findings were interpreted as complementary perspectives rather than as evidence of payer-specific saturation or definitive subgroup patterns.

Some quotes presented in the *Results* section were lightly edited for length and clarity while preserving participants’ original meaning.

### Ethical Considerations

The University of Wisconsin-Madison Health Sciences Institutional Review Board determined that this project constituted quality improvement activities and did not meet the federal definition of human subjects research. Although the project was not classified as human subjects research, a verbal consent form was reviewed with each participant prior to starting the interview. The consent provided information about the study’s purpose, the voluntary nature of the study, the risks associated with the study, protection of identity, and how the interview information would be used.

## Results

### Overview of Findings

Decision-making contexts varied across differing governance structures in the organizations represented in this study. The health system participants described decision-making occurring in a matrix organizational structure requiring perspectives across departments and roles rather than relying on a single decision-maker. Conversely, the provider-owned health plan payer described an initial decision-making process with internal leaders, followed by a subsequent request for partnership with their affiliated health systems. The Medicaid payer described decision-making guided by federal and state needs, with evaluation often provided by external partners.

**Figure 2.**
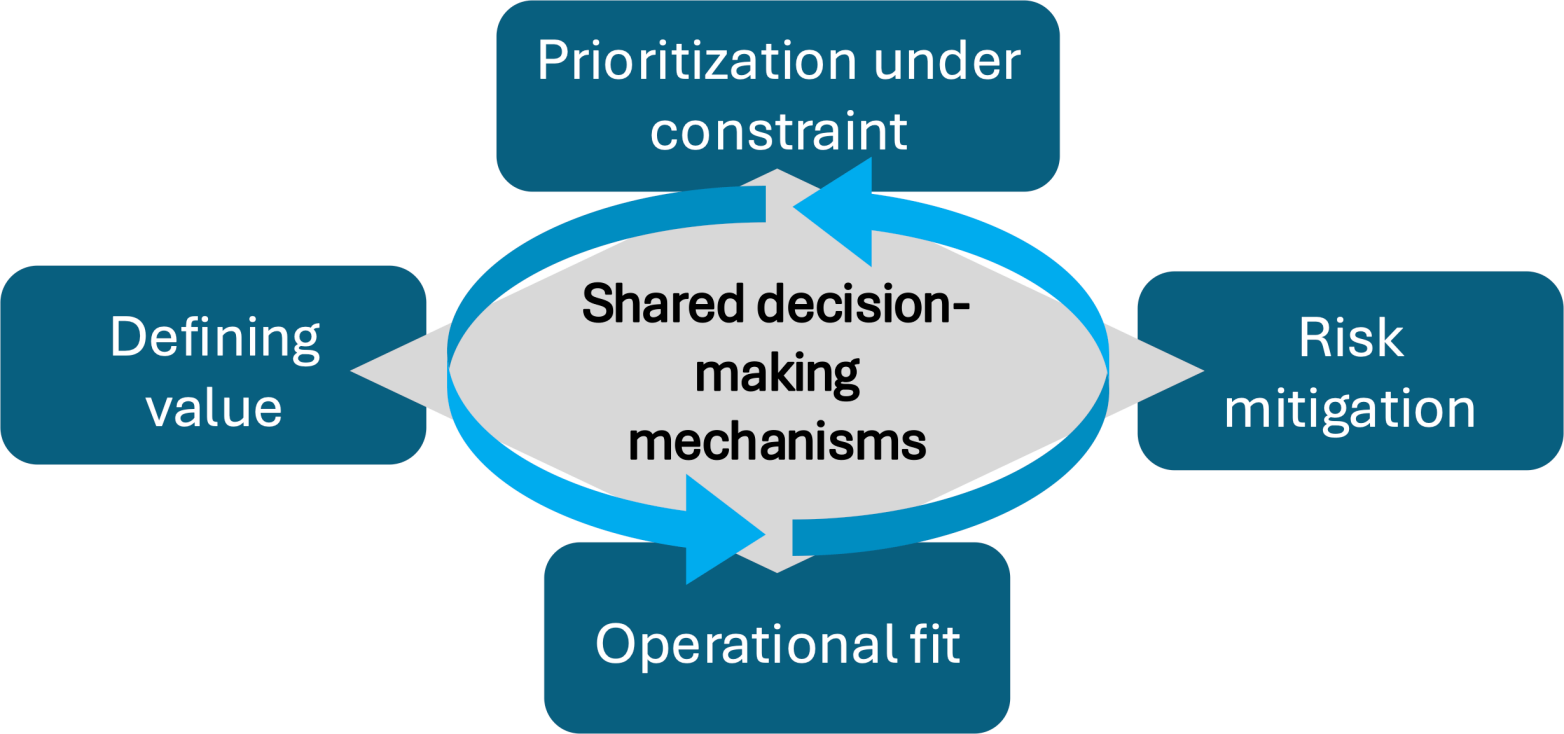
Organizational decision-making mechanisms influencing adoption and sustainability of patient-facing digital health tools. Arrows indicate their iterative and interconnected relationships.

Four themes captured shared patterns in health system and payer adoption and sustainability decision-making: prioritization under organizational constraint, risk mitigation, operational fit, and value determination. Across these mechanisms, participants evaluated patient-facing digital health tools not only for clinical merit but also for the technological demands required to support them over time. These mechanisms appeared in both health system and payer interviews, but participants emphasized different aspects of these mechanisms based on their organizational roles. Because only 2 payer leaders were interviewed, these differences in emphasis should be interpreted not as evidence of definitive convergence or divergence between groups. Figure 2 provides a visual summary of these interconnected mechanisms.

### Prioritization Under Organizational Constraint

Participants described decision-making occurring in environments characterized by limited organizational capacity and a high implementation demand for digital health tools. Constraints included financial resources, staff time, technical support, and leadership attention. Within these constraints, prioritization served as a required and ongoing process to determine which initiatives could realistically be adopted and whether a tool continued to receive organizational support over time. As one health system leader noted:

*If something looks like it’s not going to get over the finish line, we need to say, we’re done. Until you have a clinical champion, adequate resourcing, and a clear workflow change, we’re not investing technical time. We have 2,000 things we’re supposed to be considering at any given time. We have to close out a lot of ideas simply because we don’t have the time or capacity to support them*.

[HSL1]

During initial adoption decisions, prioritization was informed by population health needs, perceived impact or reach of the tool, and alignment with organizational goals and mission.

*If an initiative targets a high-priority issue for the state, like maternal mortality or reducing disparities, and we don’t think there is potential harm, we may decide it’s worth pursuing even if there isn’t yet a lot of data.*

[PL1]

*Adoption depends on alignment with both our mission-driven strategies and shorter-term priorities, but tools that align with the organization’s longer-term direction tend to be more sustainable.*

[HSL1]

Clear ownership and accountability over the tool were crucial to prioritization, with leaders distinguishing between formal ownership and clinician championship. Formal ownership involved responsibility for clinical use, data, and technical maintenance, while clinician champions were responsible for initiating, promoting, and maintaining use of the tool. Without either role, participants noted more resistance to prioritizing the tool.

*With digital applications, the key question is always who the clinical lead is and who is accountable for the care happening through the app. Sometimes it is embedded within the health system care team, and sometimes it is contracted out, but that accountability has to be clear.*

[PL1]

This same clear ownership and accountability criteria also persisted beyond adoption and operated in sustainability decisions. In the event of a loss of a formal owner or team responsibility for a tool’s oversight, leaders reevaluated tools to determine if they could still be prioritized under limited resources.

*We’ve run into situations where a tool needs support long after the person who developed it left the organization. Then the question becomes: do we still need it, does it need updating, and do we even have the resources to support it? What starts as a technical question often turns into a resource discussion.*

[HSL2]

In these sustainability evaluations, leaders also noted that a tool must remain aligned with organizational goals or a population health need to be maintained.

*At that point, it really comes down to prioritization. We look at the data and ask whether this still aligns with our priority populations and issues.*

[PL1]

Overall, prioritization emerged as a dynamic process shaped by limited resources, strategic alignment, and clearly defined accountability.

### Risk Mitigation

#### Overview

Participants described reducing risk as an important consideration when evaluating patient-facing digital health tools. Risk for patient-facing digital health tools encompassed concerns surrounding patient safety, clinical efficacy, data privacy and security, protection of organizational resources, and avoidance of unnecessary costs. To manage these concerns, leaders mentioned several strategies used to minimize risk both before and after organizational adoption. Notably, conversations about risk were concentrated among digital roles within the health system and payer leaders, due to their role-related responsibilities, which include direct oversight of vendor contracts, finances, and data security.

#### Preadoption Risk Management

Prior to adoption, leaders described several strategies to reduce risk. One strategy included piloting the tool at a smaller scale or using a time-constrained trial to determine if broader adoption was appropriate.

*Sometimes a request comes in where we’re not sure we want to use the technology yet, but we want to try it. For a limited period of time, we’re allowed to use a tool just to give feedback, and if we like it, it may result in a more formal contract that we would then pay for.*

[HSL2]

Participants also described evaluating vendors prior to adoption, including reviewing existing evidence, assessing market maturity, examining other adopters, and incorporating contractual safeguards, such as performance guarantees, to shift some of the risk related to costs and outcomes onto the vendor.

*If you don’t have the evidence, that’s really risky for us, so we’re not going to pay high dollar for it. We want it priced lower, and if it doesn’t pan out, we want the vendor to take on some of that risk. Within the contract, we look for performance guarantees, like reporting being on time and accurate or expectations around enrollment in the first year.*

[PL2]

*We also look to see who else is an adopter, even an early adopter of the partnership. We often won’t sign on unless they’ve been in market for, like, two or three years and have some early outcomes on engagement. We want to see whether they’re doing their own research to continue to validate and iteratively improve the experience before we sign on.*

[PL2]

Additionally, leaders described performing privacy and security assessments of the technology, such as how data is shared and stored, as a crucial component of risk evaluation in adoption decision-making.

#### Variation in Risk Tolerance

The level of risk considered acceptable varied. Some participants focused on cost, explaining that although money was often a concern, certain risks were worth taking if the investment seemed justified. In other cases, a Medicaid leader described being more open to piloting newer tools if they aligned with urgent state priorities and posed minimal potential harm:

*If this is a target initiative running a very important issue that’s high priority for our state... even if there’s not yet a lot of data around it, and we don’t think there’s potential harm, then... we should consider it, especially as part of this kind of pilot program.*

[PL1]

These perspectives illustrate how risk tolerance may shift depending on whether decisions are framed as financial investments or population health priorities.

#### Postadoption Risk Management

Following adoption, leaders described the continued utilization of methods to monitor and manage risk. The level of oversight of tools often reflected the extent of the financial or operational demand, with more expensive or resource-heavy tools under more scrutiny than lower-cost tools.

*Some of the ones that are more expensive for us to pay for, we have a more consistent approach to continuing to re-evaluate that vendor relationship and the technology, to make sure we’re getting the most out of the money we’re spending.*

[PL2]

Vendor relationships were also viewed as an ongoing source of risk, particularly for third-party tools. Leaders emphasized the importance of responsiveness to technical issues or continued maintenance of the tool, which demonstrated the vendor’s ability to manage risk on behalf of the organization.

*If it’s a third-party tool provided by a vendor, we have to consider the relationship. From an IT perspective, do we have a good relationship with them? When issues come up, do they actually fix them, or are we constantly working through problems that never get resolved?*

[HSL3]

In sum, risk mitigation influenced both initial adoption and long-term sustainability of a tool, with oversight and the acceptable level of risk varying in proportion to cost or strategic alignment.

### Operational Fit

#### Overview

Participants described operational fit as both an adoption and sustainability decision-making criterion defined as a tool’s ability to integrate into existing administrative, clinical, and technical operations rather than simply be made available. Participants emphasized that tools must not only establish operational fit at adoption but also maintain that fit to be sustained.

#### Clinical Workflow Integration

Seamless integration of a patient-facing digital health tool into clinical workflows was described as a significant component of operational fit to avoid adding cognitive and time burden on providers as well as facilitate utilization in an already constrained environment. Leaders highlighted that even well-integrated tools must be justified by benefits to the provider or patient to offset the additional time required by clinicians to use the tool in clinical practice.

*The way we make the decision is there has to be this kind of cost–benefit. If you can provide patients with tools that actually work, that has real benefit. So, figuring out how to make it part of the workflow means that even if it adds a little more time up front, it still pays off.*

[HSL4]

#### Technical and Administrative Integration

Operational fit was also defined as a decision-making consideration shaped by the extent to which digital tools create operational burden. Health system leaders noted favoring tools that reduced redundant manual work, while payers noted attention to payment structures for tools that reduced administrative burden.

*The most important part of technology is reducing redundant manual work so people can work at the level of their license.*

[HSL1]

*Then we start to look at, well, what does contracting look like? ... paying via a claim is way easier than having to be invoiced. Anything we can do as processing as claims saves that administrative budget.*

[PL2]

Participants also favored tools that could integrate with existing technologies rather than acting as standalone systems, mentioning benefits to clinical workflows and reduced fragmentation. With this, data integration also shaped adoption decisions based on whether the data generated from the digital health tool could be seamlessly embedded into clinical care or the electronic health record.

*There are clear benefits to building on existing systems rather than introducing a standalone tool. Having that kind of integration is really important, both for the clinical side and the IT side.*

[HSL3]

*They [vendors] might have great patient tools and a really awesome app, but the way they share data on the backend is awful. There’s mismatched data, they’re not correcting the right fields, and they don’t have it automated.*

[PL2]

#### Sustaining Operational Fit

After adoption, operational fit also had to be maintained to warrant continued use. Participants did not describe a single standardized process for maintaining operational fit across all tools. Instead, sustaining fit appeared to depend on continued organizational support after adoption. This included clear ownership and accountability for the tool, as described under prioritization, and more formal reevaluation for tools with substantial cost or resource demands, as described under risk mitigation. More directly, participants described operational fit as being maintained through continued visibility and workflow feedback.

*The app has to become relevant. You have to message it and advertise it, so it stays in people’s consciousness for them to use it.*

[HSL5]

*If you don’t give clinics or providers feedback on their own data, things can slip over time. Having reports helps ensure workflows are being carried out as intended.*

[HSL6]

Sustained operational fit of a tool is also contingent on its ability to keep up with the evolution of technology, patients’ expectations of the technology, its integration with existing technologies, or reassessing when there is overlapping functionality between 2 different systems.

*You have to keep evaluating, okay, now we’re working with two systems. Does it make sense to migrate into a more integrated system because it’s easier and more efficient to manage one system? Those are things you have to keep evaluating from a technology landscape perspective, like what your options are.*

[HSL3]

Overall, operational fit influenced both adoption and sustainability decisions, with tools required to integrate seamlessly, minimize burden, and adapt over time to remain viable.

### How Value Is Defined and Evaluated

#### Overview

Participants described 2 forms of value associated with patient-facing digital health tools: perceived value and demonstrable value. Perceived value included hypothesized benefits that generally sparked initial interest in a tool, while demonstrable value included measurable outcomes that could more directly inform adoption and sustainability decisions.

In practice, these forms of value appeared to operate together. For example, 1 payer described adopting a digital tool for a certain condition, which was initially attractive because it could expand access to services that were not equitably available across the existing care network. However, despite strong engagement, the tool became difficult to sustain when use continued longer than expected, costs accumulated, and return on investment (ROI) was not demonstrated over time. This example illustrates how perceived value could support initial investment in a tool, while demonstrable value shaped whether that investment could be sustained.

#### Perceived Value of Digital Health Tools

Perceived value was described as the anticipated or expected benefits of a digital health tool rather than the measurable benefits. This type of value was not sufficient to justify adoption but justified whether the tool solicited piloting, more exploration, or formal evaluation for adoption.

Components of perceived value included features of the tool that could offer upstream disease prevention efforts, support patient-centered care, expand the set of resources clinicians could offer patients, and expand patient access to treatment options. For example, interviewees stated:

*If patients had a digital tool, they could manage themselves, they could track things like risky behaviors or mood. That knowledge and awareness would give them power when they’re ready to have a conversation.*

[PL2]

*We don’t have enough behavioral health and primary care workforce to serve the population, especially for Medicaid. These apps could, if done well, help close some of those gaps.*

[PL1]

#### Demonstrable Value of Digital Health Tools

As opposed to perceived value, participants described demonstrable value as tangible metrics that measure the value, outcomes, efficiency gains, or ROI of a tool, noting that the types of metrics used to evaluate a tool may vary depending on the goals of the tool or organization. For example, a payer noted that when a tool was provided at no cost, evaluation focused primarily on patient outcomes rather than financial return, demonstrating how definitions of demonstrable value are variable depending on the context. Importantly, a tool is required to clearly demonstrate this type of measurable value to be considered for adoption or sustainability.

Both health system and payer leaders emphasized that tools that provide reductions in high-cost utilization, such as reductions in emergency department visits, hospitalizations, or expensive medications, are indicators of demonstrable value.

*We still have to prove value or return on investment. With population health investments, we know there’s usually a three- to five-year window before you start seeing money back. Early on it may cost more, but over time we look for reduced high-cost utilization, like fewer emergency visits, inpatient admissions, and less use of expensive medications.*

[PL2]

In addition, participants emphasized clinical effectiveness as an indicator of value, captured through both objective and subjective measures. Objective measures, such as evidence-based effectiveness data or improvements in clinical metrics (eg, reductions in AUDIT-C scores) [33], were required to validate clinical effectiveness in adoption decisions. Patient-reported subjective outcomes functioned as complementary to objective measures in decision-making and included anecdotal improvements in quality of life or disease outcomes.

*If you’re using the AUDIT-C or something like that, I think that’s one of the things the institution would look for, a decrease in something measurable.*

[HSL7]

*We’re interested in patient-reported outcomes and more global well-being outcomes, not just what happened in the medical setting.*

[PL1]

Operational efficiency also functioned as a marker of value, particularly when tools reduced administrative workload or redundant tasks.

*From a health system perspective, we look at whether a tool creates operational efficiencies, like fewer calls to the scheduling or access center.*

[HSL3]

Patient engagement was described as a challenging yet still relevant indicator of demonstrable value. Leaders defined engagement in terms of patient utilization of the tool or their engagement with the health system; however, they emphasized that engagement with digital tools can be difficult to measure and interpret in ways that inform adoption decisions.

*People look at engagement—how often someone is logging in, completing surveys, or whether it’s informing how they engage in healthcare.*

[PL2]

*Everyone understands the concept of patient engagement, but we don’t really have a historical discipline for measuring what that engagement looks like or making decisions off of it. That tends to raise a lot of questions when we’re trying to evaluate new digital tools.*

[HSL3]

On a broader level, difficulties were described in demonstrating the overall demonstrable value of patient-facing digital health tools within differing reimbursement models. The value of clinical improvements within a value-based care system was often difficult to communicate in a prevailing fee-for-service mentality that values short-term ROI or revenue.

*It’s been very difficult to make a case with our finance team about the impact, because they’re saying we won’t be bringing in new revenue... even though we might be offsetting costs... If we can’t directly show the long-term impacts of that, we’re not actually bringing in new revenue.*

[HSL6]

Overall, participants described value as a decision-making process: anticipated benefits could justify evaluating or piloting a tool, but continued support depended on whether those benefits could be measured in ways that mattered to the organization.

### Summary of Decision-Making Themes

Together, these 4 themes illustrate how participants described adoption and sustainability decisions for patient-facing digital health tools. Implicit within these themes are the underlying questions that are considered in adoption and sustainability. These questions are summarized in Table 2.

**Table 2.** Implicit decision questions shaping adoption and sustainment of patient-facing digital health tools.

Decision-making mechanism	Implicit decision question
Prioritization under organizational constraint	Given our limited capacity, resources, leadership attention, and available ownership, is this something we can realistically take on or continue supporting?
Risk mitigation	What steps are needed to reduce uncertainty and protect against clinical, financial, or operational risk before adoption, and how much ongoing scrutiny is required once the tool is in use?
Operational fit	Can this tool be implemented within existing clinical workflows, administrative processes, and technical systems at adoption and maintain that fit over time as systems evolve?
Value determination (perceived and demonstrable)	What anticipated benefits does this tool offer, and what measurable clinical, financial, or operational outcomes does it demonstrate to justify adoption and continued investment?

## Discussion

### Principal Findings

This study identified 4 decision-making mechanisms that shaped patient-facing digital health tool adoption and sustainability decisions among the health system and payer leaders interviewed. These processes demonstrate how leaders navigate constraints, risk, operational compatibility, and value determination within real-world contexts. The interaction among these mechanisms suggests that decisions were not made through a linear evaluation of whether a tool was effective or useful. Instead, participants described a process in which each mechanism shaped how the others were interpreted. A tool’s perceived value could make it worth exploring, but prioritization determined whether the organization had capacity to support it, risk mitigation shaped what safeguards were needed, operational fit determined whether the tool could be integrated into practice, and demonstrable value influenced whether support could be continued over time. This helps explain why patient-facing digital health tools with evidence of benefit may still struggle to move into sustained organizational use.

Prioritization, risk mitigation, operational fit, and value determination are not unique to digital health but reflect broader organizational challenges relevant to implementing many new health interventions. In this study, however, these mechanisms were shaped by digital health-specific concerns, including technical integration, data sharing, privacy and security review, vendor reliability, software maintenance, and interoperability.

By showing how these digital health-specific concerns shape broader implementation considerations, this study builds on prior work that examined implementation of a digital health tool at the clinical level, which framed how clinic managers, clinicians, and patients weighed perceived gains and losses to assess a tool’s adoption value [34]; the present study advances the literature by moving upstream to examine how these trade-offs function within organizational and payer-level decision processes and governance structures.

At the broader organizational level, previous work has also identified areas of alignment and misalignment across various stakeholder organizations involved in patient-facing digital health tool adoption. For example, health systems and payers have both focused on internal processes such as workflow fit and operational integration, even though they may represent separate adoption pathways [2,22]. The current study extends this work by illustrating how similar considerations in decision-making processes appear across these organizations. Health system interviews often emphasized internal governance, clinical ownership, workflow integration, technical support, and organizational capacity, whereas payer interviews more often emphasized coverage and reimbursement structures, quality metrics, and population health priorities. These differences in emphasis may point to important areas for enhanced collaboration between the 2 groups at the level of their shared decision-making processes and differences in emphasis, which complement prior work calling for a more collaborative and patient-centered digital health ecosystem [2].

While various frameworks offer a structured approach to evaluating patient-facing digital health tools across domains such as effectiveness, data, usability, privacy and security, and value [12,14-17], the findings of this study suggest that evaluation is better understood not as a static checklist but as a dynamic process of adapting decisions and organizing priorities. This perspective reflects a systems-informed view of implementation, in which adoption decisions occur within the context of organizational structures, stakeholder relationships, and competing operational priorities. Such an interpretation aligns with broader health care leadership and decision-making literature that highlights the limitations of purely rational decision models in health systems, emphasizing that leaders operate in continuously transforming systems requiring adaptation and broad strategies, rather than relying on uniform criteria [35,36]. Taken together, this study illustrates how and why digital health tool evaluation frameworks may be incomplete because they account for what should be assessed but do not capture how organizational leaders actually weigh, sequence, and adapt those criteria in practice.

### Implications

These findings suggest that evidence of organizational benefit or fit is a necessary but not sufficient criterion for adoption and sustainability of patient-facing digital health tools. Instead, tools are evaluated within a broader prioritization process that requires alignment with organizational goals, resource availability, and current population health prevalence amidst an overall high technological implementation demand. This framing illustrates how digital tools are assessed in relation to competing initiatives rather than on their own merits. As a result, tools may not be adopted or sustained if they cannot be prioritized within existing organizational capacity and competing demands.

The findings related to navigating risk exposure highlight the importance of protecting both patients and the organization, especially in resource-constrained environments as mentioned above. Risk, therefore, did not have to function as an organizational barrier to adoption due to strategies such as pilots, contractual safeguards, privacy and security assessments, and signals of vendor maturity that were used to manage risk-related concerns. Additionally, risk mitigation extended beyond adoption, with ongoing attention to vendor responsiveness and greater oversight for tools requiring substantial organizational resources. Adoption decisions for patient-facing digital health tools therefore depend less on eliminating uncertainty altogether and more on whether organizations have feasible strategies to manage and monitor both the clinical and technological risks over time.

Operational fit occurred when a tool could be integrated into existing workflows, reduced redundant work, and aligned with current data systems. Sustainability, in turn, relied on whether this fit could be maintained over time through continued visibility, feedback loops to clinicians, and technological relevance, rather than clinical effectiveness alone. These findings indicate that operational compatibility of a tool must be demonstrated both prior to adoption and through the ability to maintain that fit over time as workflows, technologies, and organizational needs evolve. Sustainability planning should also include outlining processes for monitoring this fit.

The distinction between perceived and demonstrable value helped explain how adoption and sustainment decisions were justified. Perceived value could move a tool into further evaluation or pilot testing when it appeared likely to address an important organizational need, but those early assumptions had to be translated into demonstrable value outcomes to support continued investment. For patient-facing digital health tools, this distinction was especially important since value depends not only on whether patients are engaging with the intervention but also on whether that tool could generate improved outcome metrics that an organization can measure. This suggests that developers, researchers, and organizations should plan for demonstrable value early by defining and clearly demonstrating what evidence would be needed to determine whether a tool should be sustained. Additionally, the indicators of value used in decisions were often a holistic, context-dependent process. Metrics were weighed differently depending on the goals of the tool and reimbursement context used, including trade-offs between longer-term value-based care benefits and shorter-term fee-for-service gains. Together, this illustrates that value is not a fixed criterion but varies in how it is defined and applied based on the needs and priorities shaping the decision.

Together, these findings imply that continuous challenges in the implementation of patient-facing digital health tools within health system and payer organizations may arise from the complex ways in which prioritization, risk management, operational fit, and value determination interact within constrained decision-making environments. Making these decision-making processes visible can help better align digital health developers, researchers, and policymakers with the realities of health care environments.

### Limitations

This study was conducted within a single health system organized in a matrix governance structure that includes an accountable care organization operating with capitated and value-based care arrangements. As a result, findings may not be generalizable to other health systems with different governance structures or payment models. However, the main themes of prioritization, risk mitigation, operational fit, and value determination are likely to arise in other organizations evaluating patient-facing digital health tools, though their relative importance may vary by setting, such as in smaller, less integrated, rural, or safety-net settings, for example.

The payer perspective was represented by both a Medicaid and a provider-owned health plan leader, which limits the depth of comparative analysis between health system and payer decision-making. Although payer interviews provided important insight into coverage, reimbursement, quality, financial, and population health considerations, the small payer sample limits our ability to determine whether the observed differences in emphasis represent broader convergence or divergence between health system and payer decision-making. Additionally, because this study also included a larger number of representatives from the health system (n=7) in proportion to the number of representatives from payer organizations (n=2), findings may have resulted in greater depth in the health system perspectives. Additional research with a larger and more diverse payer sample is needed to determine how adoption and sustainability decisions may differ across payer types or from health system decision-making.

Additionally, because the Tula app was used as an illustrative use case, AUD-specific considerations such as stigma, treatment access gaps, fragmented care pathways, and AUDIT-C outcome measurement informed parts of the interviews. However, these considerations were reflected within the 4 broader mechanisms rather than forming separate themes. Findings may therefore be most transferable to patient-facing digital health tools that require similar organizational decisions, while tools for other conditions may raise different considerations.

Finally, although all of the organizations represented in this study host patient-facing digital health tools (eg, patient portals and electronic health record–integrated tools), there was variation in experience hosting standalone patient-facing mHealth apps, like Tula. The provider-owned health plan had prior implementation experience with such tools, whereas the health system and Medicaid organization did not. As a result, some adoption and sustainability considerations may reflect differences in implementation experience with different types of patient-facing digital health tools.

### Future Directions

Although outside the scope of the primary analytic focus, several participants provided insight into how their organization addresses emerging technologies, including patient-facing AI tools. These discussions implied that patient-facing AI was considered in alignment with the existing decision-making processes described throughout this study (prioritization, risk management, operational fit, and value determination). Notably, particular scrutiny was centered on risk, with concerns related to misinformation, liability, or unclear regulatory oversight. As a result, limiting patient-facing AI initiatives to lower-risk uses, such as billing assistance, was described as a current approach to manage some of that uncertainty. Overall, this suggests that as patient-facing digital health tools begin to integrate AI modalities, decision-making processes may require higher scrutiny and additional complexities related to governance and risk.

## Supplementary material

10.2196/97536 Multimedia Appendix 1 Example semistructured interview guide.

## Abbreviations

AUD: alcohol use disorder
AUDIT-C: Alcohol Use Disorders Identification Test-Consumption
mHealth: mobile health
ROI: return on investment
