# Supplementary material for: Health System- and Payer-Level Decision-Making Processes Influencing the Adoption and Sustainability of Patient-Facing Digital Health Tools: Qualitative Study
Source: J Med Internet Res. 2026 Aug 14;28:e97536. doi: 10.2196/97536 (PMC13476001; doi:10.2196/97536)
Supplement: Supplementary file 1 [file jmir-v28-e97536-s001.docx]

# **Multimedia Appendix 1. Example Semi-Structured Interview Guide**

### 1) Role & Organizational Context

**Purpose:** To understand the participant’s role, decision-making authority, and how digital health fits within organizational priorities and governance structures.

- Could you tell me a bit about your role and how it relates to technology and digital health?
- When new tools are considered, how do clinical leaders, IT, operations, or other teams work together in the decision-making process?
- How easy or challenging is it to pilot and scale new tools across the organization?

### 2) Current AUD Management Landscape

**Purpose:** To understand how Alcohol Use Disorder is currently managed or supported and where gaps or challenges exist.

- How is Alcohol Use Disorder currently managed or supported within your organization?
- Where are the biggest hurdles whether related to screening, access to treatment, coordination, or keeping patients or members engaged over time?
- What challenges exist related to coverage, clinician capacity, coordination between behavioral health and primary care, or member engagement?

3) Digital Tool Adoption Decisions

**Purpose:** To understand how new digital tools are reviewed within organizations and what factors influence whether a tool is adopted, supported, or covered.

- When a new digital tool is introduced, who is typically involved in deciding whether it gets adopted, supported, or covered?
- What criteria matter most when evaluating a tool — such as ease of use, fit with workflow, cost, impact on patients or members, or alignment with organizational goals?
- How do you define “evidence-based” when assessing a digital tool or treatment?
- With so many digital tools on the market, what helps one stand out or move forward within your organization?

### 4) Value & Fit of Tools Like Tula

**Purpose:** To understand how organizations define value and what makes a digital tool feel worth adopting, supporting, or covering.

- When you think about the “value” of a digital health tool, what comes to mind — return on investment, helping patients or members, saving staff time, or something else?
- What kinds of outcomes matter most — better engagement, fewer hospital visits, cost savings, equity impacts, or quality metrics?
- What makes a digital tool feel worth adopting, supporting, or covering within your organization?
- How important is integration with existing systems or workflows in determining whether a tool feels like a good fit?

### 5) Barriers to Adoption

**Purpose:** To identify common obstacles that prevent digital health tools from being adopted or implemented successfully.

- What are the biggest barriers you’ve seen when introducing or supporting new digital health tools?
- Where does resistance typically arise (i.e. from leadership, clinicians, or elsewhere)?
- Is it difficult to generate or maintain momentum without a strong internal champion?
- Are behavioral health tools, including those for AUD, especially hard to implement? If so, why?

### 6) Sustainability & Long-Term Fit

**Purpose:** To understand what allows a digital health tool to move beyond pilot stages and remain supported over time.

- What helps a digital tool move from a short-term pilot to long-term adoption or support within your organization?
- What kinds of evidence or outcomes matter most when deciding whether to continue funding, covering, or using a tool?
- What tends to cause digital tools to fade out after initial rollout or pilot phases?
- What keeps a tool useful in day-to-day practice over time?

### 7) Closing Reflections

**Purpose:** To gather broader reflections on digital health decision-making and recommendations for researchers and developers.

- What advice would you give to researchers or developers building tools in this space?
- What do you wish people better understood about how payers/health systems think about digital health adoption?
- Who else do you think it would be helpful for us to talk with?
